# Diversity and Dynamics of *Salmonella enterica* in Water Sources, Poultry Litters, and Field Soils Amended With Poultry Litter in a Major Agricultural Area of Virginia

**DOI:** 10.3389/fmicb.2019.02868

**Published:** 2019-12-17

**Authors:** Ganyu Gu, Laura K. Strawn, Jie Zheng, Elizabeth A. Reed, Steven L. Rideout

**Affiliations:** ^1^Eastern Shore Agricultural Research and Extension Center, Virginia Tech, Blacksburg, VA, United States; ^2^Center for Food Safety and Applied Nutrition, US Food and Drug Administration, College Park, MD, United States

**Keywords:** *Salmonella* distribution, prevalence, serovar, agricultural samples, environmental samples, foodborne pathogens

## Abstract

The Eastern Shore of Virginia (ESV) is a major agricultural region in Virginia and in the past has been linked to some tomato-associated outbreaks of salmonellosis. In this study, water samples were collected weekly from irrigation ponds and wells in four representative vegetable farms (Farms A–D, each farm paired with one pond and one well) and a creek as well. In addition, water samples from two sites in the Chesapeake Bay on the ESV were collected monthly. Poultry litter was sampled monthly from three commercial broiler farms. Soil samples were collected monthly after fertilization with poultry litter from 10 farms in 2014 and another 14 farms in 2015. A most probable number method was used to detect *Salmonella enterica* presence and concentration in collected samples. Presumptive *Salmonella* colonies were confirmed by the cross-streaking method. Molecular serotyping was carried out to determine the *Salmonella* serovars. The average prevalence of *Salmonella* in pond, well, creek, and bay water samples was 19.3, 3.3, 24.2, and 29.2%, respectively. There were significant spatial and temporal differences for *Salmonella* incidence in various water sources. The prevalence of *S. enterica* in four tested ponds from farms A, B, C, and D were 16, 12, 22, and 27%, respectively. While the prevalence of *S. enterica* in irrigation wells was significantly lower, some well water samples tested positive during the study. *Salmonella* Newport was found to be the predominant serovar isolated from water samples. All poultry houses of the three tested broiler farms were *Salmonella*-positive at certain sampling points during the study with prevalence ranging from 14.3 to 35.4%. *Salmonella* was found to be able to survive up to 4 months in poultry litter amended soils from the tested farms in 2014, and up to 6 months in 2015. This research examined the dynamics of *S. enterica* in relationship to water source, poultry litter, and amended soil in a major agricultural area, and provides useful information for food safety risk assessments.

## Introduction

*Salmonella* is the most frequently encountered bacterial pathogen associated with foodborne illness in the United States [Food Safety News (FSN), 2011; Centers for Disease Control and Prevention, 2019]. Produce has become a common vehicle for the transmission of *Salmonella*, and has been the cause of both international and multistate outbreaks in recent decades (Centers for Disease Control and Prevention, 2005, 2007; Greene et al., 2008; Bennett et al., 2015). The Eastern Shore of Virginia (ESV) has been linked to multiple outbreaks of salmonellosis from contaminated tomato fruits (Greene et al., 2008; Bell et al., 2015; Bennett et al., 2015). In each case, the same specific pulsed field gel electrophoresis (PFGE) pattern of *Salmonella enterica* Newport was implicated in the outbreak. The same PFGE type *Salmonella* Newport was also associated with the 2014 outbreak associated with cucumber, which was determined to have originated from the Eastern Shore of Maryland (Angelo et al., 2015). Therefore, Bell et al. (2015) have suggested *Salmonella* Newport may have the ability to persist in the environment (both agricultural and natural environments) in this region.

Agricultural water has been considered as a primary contamination source of foodborne pathogens during produce production, which may be contaminated by sewage overflows, polluted storm water runoff, and agricultural runoff (Solomon et al., 2002; Islam et al., 2004a,b; Hintz et al., 2010). Untreated biological soil amendments of animal origin are also potential sources of contamination of agricultural products that are consumed raw. Research has shown that plants in the field can be contaminated when foodborne pathogens are introduced by biological soil amendments (Natvig et al., 2002; Solomon et al., 2002; Ohtomo et al., 2004; Islam et al., 2004b; You et al., 2006; Franz et al., 2008a,b; Semenov et al., 2009; Park et al., 2012; Strawn et al., 2013). Poultry litter is considered to be a mix of bedding material, feathers, spilled feed, and poultry excreta, and may be used as fertilizer for crop production. With the rapid growth of the poultry industry in recent decades in the Delmarva area, farmers have taken advantage of this economical organic fertilizer. Poultry litter is applied from February through June, and applied typically in April and May on the ESV [Virginia Department of Environmental Quality (VDEQ), 2010]. Multiple researchers have shown that fresh poultry litters may be contamination reservoirs in broiler farms (Opara et al., 1992; Pope and Cherry, 2000; Van Asselt et al., 2009; Suresh et al., 2011).

It is essential to understand the dynamics and diversity of *Salmonella* in production environments to assist in development of mitigations, especially the occurrence of *Salmonella* Newport in agricultural and natural environments on the ESV. In this study, the prevalence and serovar diversity of *S. enterica* were investigated in various water sources (creek, well, bay), broiler farms (raw poultry litter), and poultry litter amended soils (in agricultural fields).

## Materials and Methods

### Water Sample Collection and Water Parameter Measurement

This study was performed on the ESV, an important agricultural region of the U.S.’s Mid-Atlantic, including Virginia’s highest tomato production county. In 2014 and 2015, 4 liters of pond and well irrigation water samples were collected weekly from four vegetable and crop farms (Farms A–D) on the ESV for *Salmonella* detection (Gu et al., 2016). On each tested farm, a pair of one pond (Ponds A–D) and one well (Wells A–D) with distance less than 500 m was selected. One week in 2014 and 3 weeks in 2015 were skipped for water sampling due to severe weather. A total of 51 and 49 weekly samples were tested for pond and well water on each farm in 2014 and 2015, respectively. One creek on Farm B was also selected for weekly water sampling from September 2014 to December 2015 with a total of 66 weekly samples. Similarly, 4-liter water samples were collected monthly from two sites of the Chesapeake Bay including one private beach in Accomack county and one public beach in Northampton county from April 2014 to March 2015 with a total of 12 samples at each site.

Water samples were placed on ice and transported to the lab for subsequent processing. A total of 400 [4 farms × (51 + 49) weeks] pond and well irrigation water samples, 66 (66 weeks) creek water samples, and 24 (2 sampling sites × 12 months) bay water samples were tested in this study.

### Poultry Litter Sampling

Three commercial broiler farms (Farm 1, Farm 2, and Farm 3) on the ESV were selected for collecting fresh and stored poultry litter samples (raw poultry litter from chicken houses in the Delmarva region were usually stored in the stacking sheds at broiler farms and applied to crop fields before the growing season). From October 2013, four chicken houses on Farm 1, three houses on Farm 2, and three houses on Farm 3 were sampled monthly for 12, 8, and 7 months, respectively. At each sampling time, 500 g of fresh poultry litter samples was randomly collected from the entire house that currently contained birds. Two sample types were included: (1) dry litter from the middle of the poultry house and (2) wet litter collected from underneath the water-dispensing lines. These two sample types will be referred to as “Dry” and “Wet” from this point forward. Stored poultry litter was also collected monthly from stacking sheds on the three broiler farms during the study.

### Soil Sampling From Farms Applied With Poultry Litter

From April 2014 to December 2014, 10 conventional farms named Farms E1–E10 on the ESV, which were fertilized with poultry litter in April of the same year, were sampled monthly for the presence of *Salmonella*. Similar soil testing was conducted in 2015. From April 2015, 14 conventional farms named Farm G1–G14 on the ESV, which were fertilized with poultry litter in April or May of the same year, were sampled monthly for *Salmonella* detection. At each sampling time, 500 g of soil was randomly collected from the 0–3 cm layer of each farm field. The soil type of sampled farms was Bojac sandy loam.

Farms tested in this study for irrigation water, poultry litter, and soil sampling were independently operated and geographically distinct (distance >3 km).

### *Salmonella* Detection and Most Probable Number Analysis

A most probable number (MPN) method was used to enumerate *Salmonella* in the water, poultry litter, and soil samples. Two different MPN schemes (I: 4 tubes × 500 ml water, 4 × 100 ml, 4 × 10 ml; II: 4 tubes × 50 g poultry litter/soil, 4 × 10 g, 4 × 1 g) were used in respective samples with a lower limit of detection (LOD; a single tube positive at the lowest concentration) of 0.41 MPN/L water, and 2.1 MPN/kg poultry litter or soil (Luo et al., 2014, 2016). MPN tubes were prepared in equal volumes of double strength lactose broth (LB, BD Biosciences, San Jose, CA) and incubated for 24 h at 37°C. Aliquots of 1 mL from pre-enriched cultures were sub-cultured to 9 mL Tetrathionate Broth (TT Broth, Dot Scientific Inc., Burton, MI) for 24 h at 37°C. Each selective enriched broth culture was then streaked onto *Salmonella*-selective xylose lysine tergitol 4 (XLT4) (BD Biosciences, San Jose, CA) agar plates and incubated for 24 h at 37°C. Presumptive-positive colonies were confirmed by the cross-streaking method using CHROMagar^™^ *Salmonella* plates (DRG International Inc., Springfield, NJ). MPN calculations were performed using the MPN calculator build 23, created by Mike Curiale. Confidence intervals were derived from the Fisher’s method, as reported by Hurley and Roscoe (1983). Up to four confirmed colonies from each positive plate were stored in brain heart infusion (BHI) broth containing 20% glycerol at −80°C.

*S. enterica* serovar Newport strain J1892 isolated from produce-associated outbreaks was used as positive control. Sterile enrichment media were used as negative control. This study was performed by following the biosafety standard operating protocols approved by the Institutional Biosafety Committee at Virginia Tech (Permit No.: IBC # 17-051).

### Weather Information Collection and Analysis

HOBO Micro Station (Onset Computer Corporation, Bourne, MA) was installed at Farms A–D. Temperature (°C) and rainfall data (mm) during the collection period were recorded for further analysis.

### Molecular Serotyping Analysis

Up to two stored *Salmonella* isolates from each positive plate were selected for serotyping analysis using the Centers for Disease and Control (CDC) standard protocol for the molecular serotyping of *Salmonella* spp. (Fitzgerald et al., 2007; Centers for Disease Control and Prevention, 2009; McQuiston et al., 2011). Briefly, DNA from a pure bacterial culture was isolated using InstaGene^™^ Matrix (BioRad, Hercules, CA). Multiplex PCR was set up using Qiagen HotStar Master Mix (Qiagen) and 1 μl of DNA, and thermocycled with the following protocol: 95°C, 15 min; 30 cycles at 94°C for 30 s, 48°C for 90 s, 72°C for 90 s; then 72°C for 10 min (Gu et al., 2018). Primers used for PCR amplification have been reported previously (Fitzgerald et al., 2007; McQuiston et al., 2011). DNA from the PCR products was then hybridized to the specific O- and H-Ag probes on beads with strepavidin-R-phycoerythrin (for fluorescent label attachment; Invitrogen div. Life Technologies, Grand Island, NY) (Centers for Disease Control and Prevention, 2009). After incubation the samples were tested using the Bio-Plex instrument (BioRad). Positives were analyzed by the ratio of signal to noise using a negative control (no template DNA). Serotype was identified based on which antigens are positive for each isolate. A positive control (*S. enterica* serovar Typhimurium strain ATCC 14028) was included in every serotyping analysis.

### Statistical Analysis

When needed for descriptive statistics, MPN values were assigned for those samples outside the range of detection (either no tubes positive or all tubes positive.) For example, a value of zero was given to samples producing no positive tubes. And the maximum enumerable MPN was assigned to samples with all tubes positive. *Salmonella* MPN levels in pond irrigation water were log transformed using the formula log_10_(MPN + 1) to present the dynamics of *Salmonella* population density in sampled ponds for normalization. The log-transformed values were used for following statistical analyses. Effects of farm location (Farms A–D), time (12 months), and water source type (pond and well) on the population density (MPN/L) of *Salmonella* in irrigation water during the 2 years were assessed by general linear mixed models (GLIMMIX) procedure. Student *t*-tests were performed to evaluate *Salmonella* population densities of pond water samples in each farm or each month between two testing years. A Chi-square test was computed to compare *Salmonella* prevalence between pond and well irrigation water samples. Pearson’s and biserial correlation coefficients were calculated to analyze the correlations between weather parameters and *Salmonella* population and occurrence, respectively, in irrigation water. The same test was performed to evaluate the difference of *Salmonella* prevalence between the two sampling sites of the Chesapeake Bay.

GLIMMIX analysis was conducted to evaluate the effect of broiler farm location and poultry litter type (wet and dry) on *Salmonella* prevalence and population density. The same analysis was performed to assess the difference of *Salmonella* population density among the sample types (water, poultry litter, and soil) tested in this study.

Statistical analysis was performed using SAS (SAS release 9.3, SAS Institute Inc., Cary, North Carolina). Except when stated otherwise, values of *p* < 0.05 were considered statistically significant.

## Results

### *Salmonella* Prevalence and Diversity in Water

**Figure 1:**
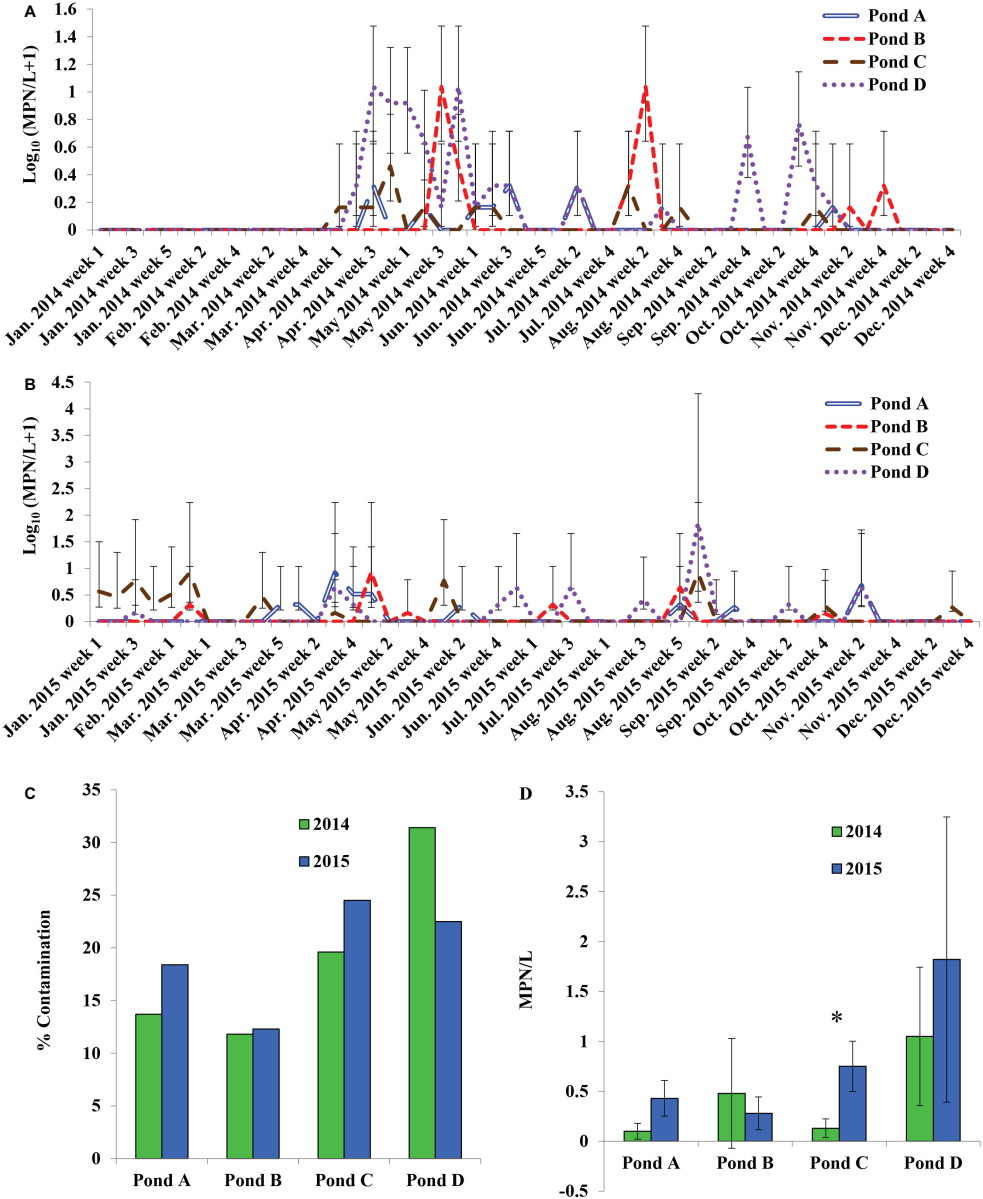
Prevalence and most probable number (MPN) values of *Salmonella enterica* in pond irrigation water of four tested farms on the Eastern Shore of Virginia (ESV). **(A)** Weekly sampling in 2014; **(B)** weekly sampling in 2015; **(C)** comparison of *Salmonella* average prevalence in ponds in both years; **(D)** comparison of average population density in ponds in both years. Bars represent 95% confidence interval in **(A)** and **(B)** and represent standard errors in **(D)**. ^*^Significant difference between two sampling years (*p* < 0.05).

**Figure 2:**
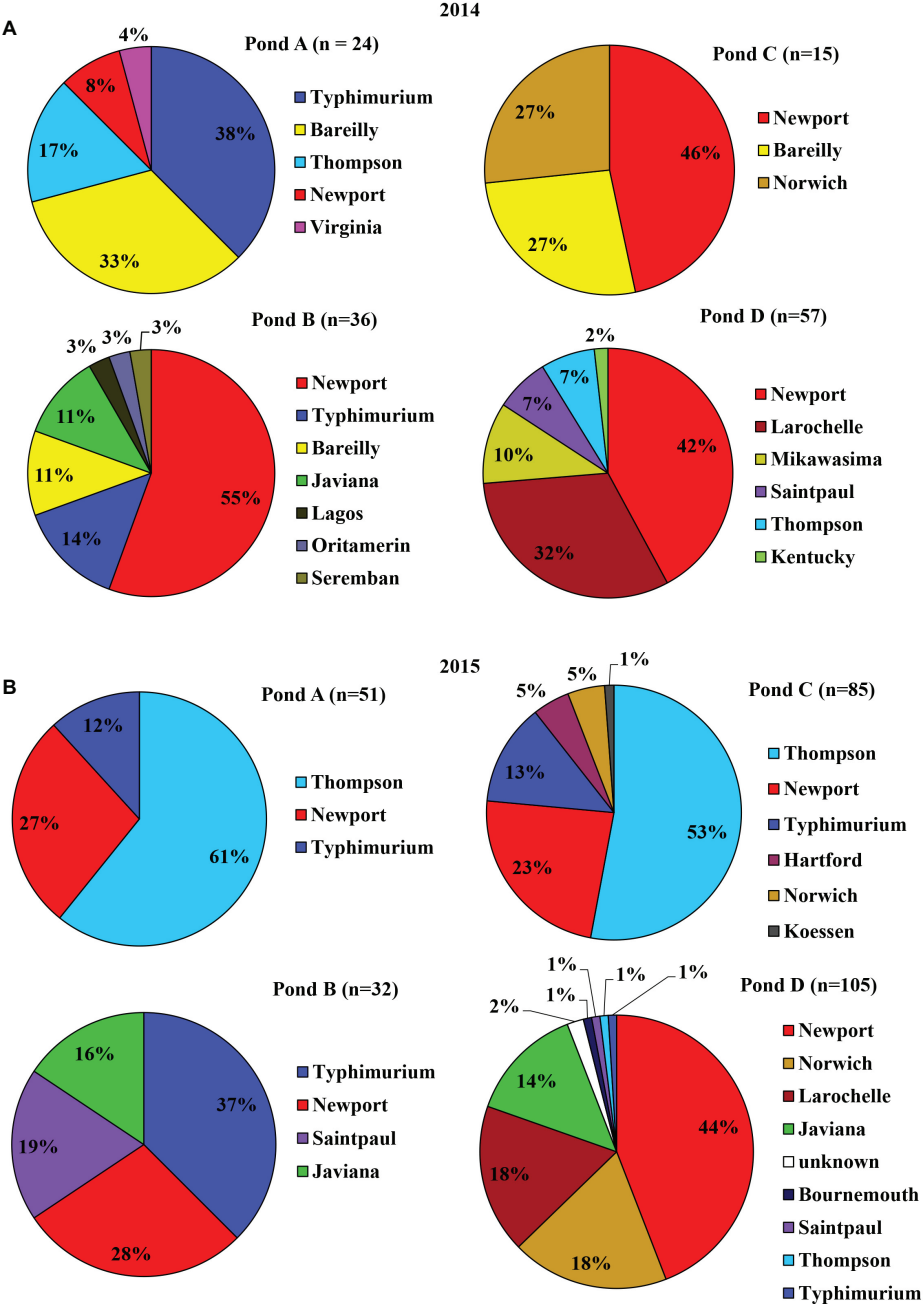
Serovar diversity of *S. enterica* isolated from surface water samples of each of the four irrigation ponds at Farms A–D in 2014 **(A)** and 2015 **(B)**. *n* denotes the number of identified *Salmonella* isolates.

The four ponds examined in this study were located on four different farms (A to D) on the ESV. All ponds were sampled weekly for approximately 2 years. There were significant interactions between sampling year and farm location for *Salmonella* occurrence and MPN values (*p* < 0.05). Therefore, effects of pond are presented separately for the 2 years (Figures 1A,B). *Salmonella* was detected in water samples from all four ponds at varying times during the course of the study [19.25% positive samples (*n* = 400); average population density: 0.63 ± 0.02 MPN/L]. The occurrence in each pond was 16, 12, 22, and 27% positive samples, respectively (*n* = 100). The average level of *Salmonella* was 0.26 ± 0.09, 0.38 ± 0.16, 0.43 ± 0.13, and 1.43 ± 0.72 MPN/L respectively. Spatial and temporal variations in *Salmonella* occurrence and levels were noticed in surface water samples (Figures 1A,B). No significant difference in the occurrence or levels of *Salmonella* was observed among ponds for water samples between year 1 and year 2 (*p* > 0.05; Figures 1C,D) except for Pond C (*p* = 0.017; Figure 1D). Pond C showed a significant increase in year 2 compared to year 1. *Salmonella* was detected in 8 of the 12 months (from April to November) in 2014, and every month in 2015 (Supplementary Figure S1A). The average population density for pond water samples varied significantly among months in both year 1 and 2 (Supplementary Figure S1B). For example, the *Salmonella* levels ranged from below LOD (January, February, March, and December) to a high of 2.11 MPN/L (May) in year 1 and from 0.05 MPN/L (December) to 4.91 MPN/L (September) in year 2. In addition, *Salmonella* levels were significantly higher in January and February in 2015 compared to 2014 (*p* < 0.05). No significant differences in *Salmonella* levels were seen between sampling sites and months for both years (*p* > 0.05). *Salmonella* occurrence in Pond A was significantly correlated to Pond D in both years (*p* < 0.01), and correlation between Ponds C to D is also significant (*p* = 0.04). The correlation of *Salmonella* populations in Pond B are also significant between 2014 and 2015 (*p* < 0.01). Among the 405 confirmed *Salmonella* isolates that were recovered from pond water samples, a total of 14 serotypes were assigned in 2014 and 10 in 2015. Newport was the most frequent serotype identified from irrigation ponds B–D in 2014. In Pond A, Typhimurium and Bareilly were the most frequently recovered serotypes (71%) (Figure 2A). The most frequent serotypes identified in Ponds A–D in 2015 were Thompson, Typhimurium, and Newport, respectively (Figure 2B). Overall, all ponds supported multiple serotypes, and serotypes Newport, Typhimurium, Bareilly, Thompson, Saintpaul, and Javiana were recovered from multiple ponds in both years. Pond D had the largest diversity of serotypes (eight serotypes) in 2015; while Pond C and Pond A had the least (three serotypes) in 2014 and 2015, respectively.

Wells examined in this study were located on the same corresponding farms as the ponds (A to D) on the ESV. All four wells were sampled weekly for approximately 2 years. Therefore, well effects are presented separately for the years studied (Table 1). Overall, *Salmonella* was detected in water samples from all wells, except for Well B, at some time point during the sampling [3.25% positive samples (*n* = 400); 0.04 ± 0.04 MPN/L] (Table 1). The prevalence and levels of *Salmonella* in well water were significantly lower compared to pond water (*p* < 0.01). A total of 49 confirmed *Salmonella* isolates were recovered from well water samples, 25 isolates in 2014 and 24 in 2015. Serotype Newport (65%, *n* = 25) represented most *Salmonella* isolates identified from well water samples in 2014, followed by Thompson (31%) and Oritamenrin (4%). Furthermore, serotype Newport was recovered from all three wells which tested positive for *Salmonella*. In 2015, Thompson (33%, *n* = 24) was the most frequent serotype identified from well water samples, followed by Typhimurium (17%) and Javiana (17%). *Salmonella* Newport was only identified from Well C (12%). *Salmonella* population and occurrence in ponds and wells were not significantly correlated with temperature and total rainfall at sampled farms in both years (*p* > 0.05).

**Table 1:** Prevalence and most probable number (MPN) values of *Salmonella enterica* in well water of the four tested farms on the Eastern Shore of Virginia (ESV) in 2014 (sampled 51 weeks in total) and 2015 (49 weeks).

	Sampling date	MPN/L	Upper limit^*^	Lower limit
**2014**				
Well A	11/10/2014	0.46	3.2	0.065
	12/15/2014	1.1	4.2	0.27
	12/22/2014	0.46	3.2	0.065
Well B	NA			
Well C	11/17/2014	1.1	4.2	0.27
Well D	11/3/2014	0.46	3.2	0.065
	11/10/2014	0.46	3.2	0.065
	11/24/2014	0.46	3.2	0.065
	12/1/2014	1.1	4.2	0.27
**2015**				
Well A	3/23/2015	3.4	9.3	1.3
Well B	NA			
Well C	6/1/2015	0.42	3.2	0.0055
Well D	9/21/2015	1.5	4.9	0.43
	9/28/2015	0.46	3.2	0.065
	12/21/2015	3.4	9.3	1.3

*NA, not detectable*.

* *95% confidence interval*.

**Figure 3:**
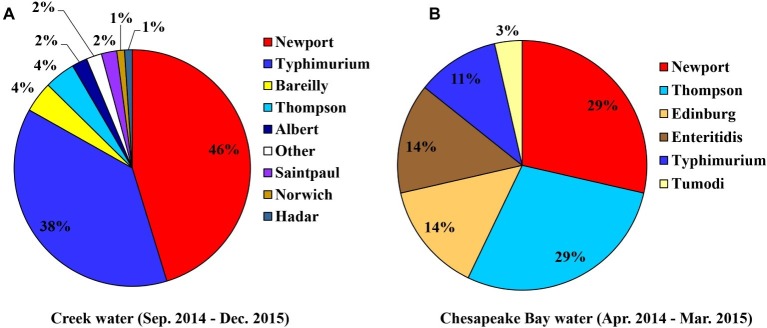
Diversity of *S. enterica* serovars isolated from sampled creek water [**(A)**, *n* = 95] and Chesapeake Bay water [**(B)**, *n* = 27].

From the creek sampled on Farm B, *Salmonella* was observed in 16 of the 66 weeks (24.2% positive samples [*n* = 66]; 1.12 MPN/L) (Supplementary Figure S2). A total of 95 confirmed *Salmonella* isolates were recovered, with eight serotypes identified by molecular serotyping. Newport was the most frequent serotype identified (46%), followed by Typhimurium (38%) (Figure 3A).

*Salmonella* was detected multiple times from the two sampling sites of the Chesapeake Bay (Table 2), five of the 12 bay water samples collected from the private beach, and two of the 12 samples from the public beach. The prevalence and average level of *Salmonella* in bay water samples were 29.2% and 0.88 MPN/L, respectively. Both Newport and Thompson were the most frequent serotypes (29%) identified from bay water samples (Figure 3B).

**Table 2:** Prevalence and most probable number (MPN) values of *S. enterica* in Chesapeake Bay water at two detection sites on the Eastern Shore of Virginia from April 2014 to March 2015 (*n* = 12/site).

	Sampling month	MPN/L	Upper limit^*^	Lower limit
Accomack County (Private beach)	April 2014	1.1	4.2	0.27
	May 2014	1.1	4.2	0.27
	June 2014	7.3	20	2.6
	November 2014	1.9	5.9	0.62
	January 2015	1.1	4.2	0.27
Northampton County (Public beach)	November 2014	0.46	3.2	0.065
	March 2015	7.3	20	2.6

* *95% confidence interval*.

### *Salmonella* Prevalence and Diversity in Poultry Litter

**Figure 4:**
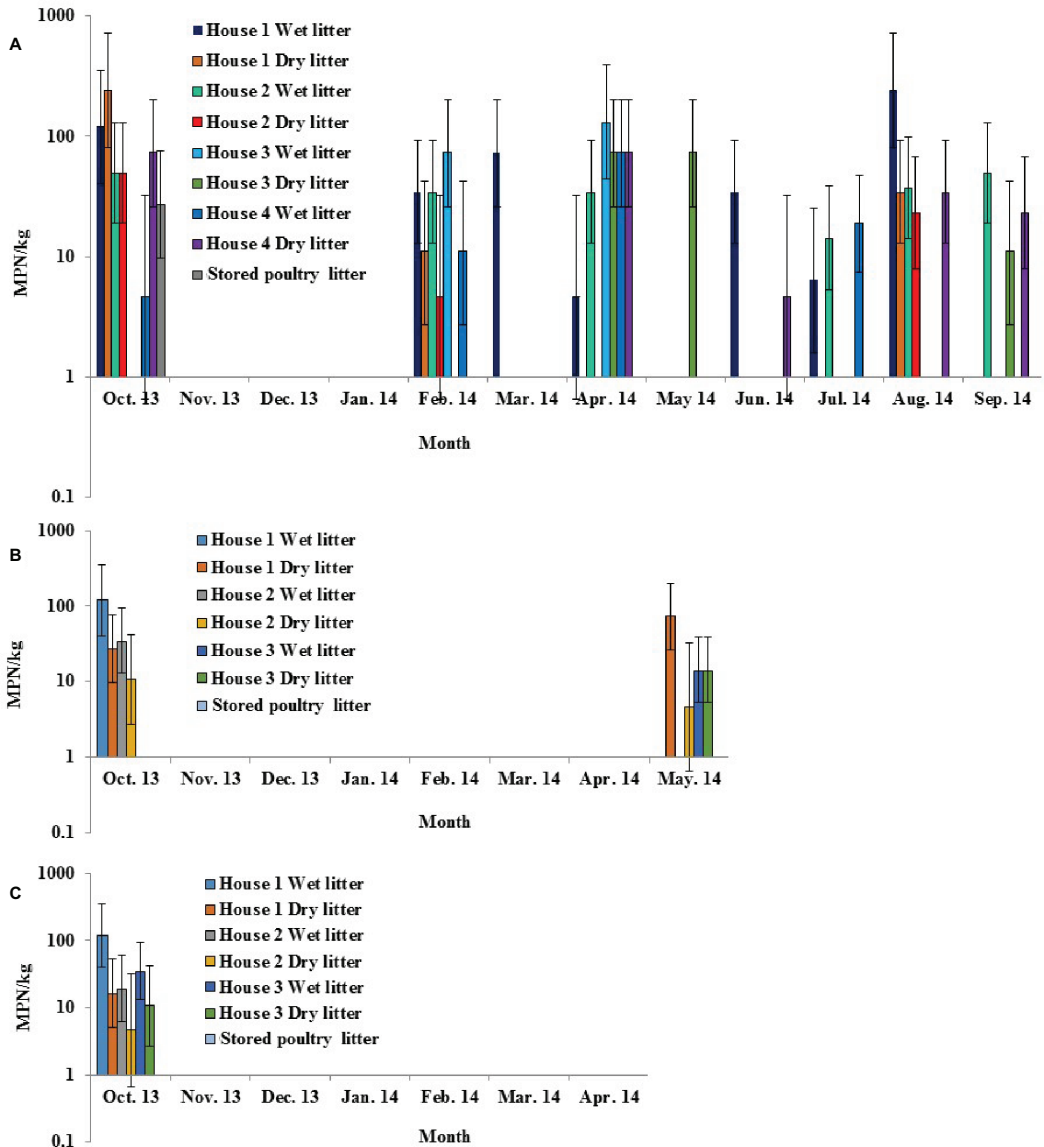
Prevalence and MPN values of *S. enterica* in wet, dry, and stored poultry litter samples collected from multiple chicken houses and stacking sheds at commercial broiler Farm 1 **(A)**, Farm 2 **(B)**, and Farm 3 **(C)** on the ESV.

All three broiler farms sampled in this study were located on the ESV. Four chicken houses on Farm 1 were sampled for 12 months from October 2013 to September 2014, three chicken houses on Farm 2 were sampled for 8 months from October 2013 to May 2014, and three chicken houses on Farm 3 were sampled for 7 months from October 2013 to April 2014. Overall, *Salmonella* was detected in poultry litter samples from all 10 chicken houses at various time points (Figures 4A–C). The overall *Salmonella* occurrence in wet (26.9%) and dry (23.7%) litter samples was not significantly different for each sampled farm (*p* > 0.05). The levels and occurrence in fresh poultry litters varied among the farms, with broiler Farm 1 (18.4 MPN/kg and 35.4%) significantly higher (*p* < 0.05) than Farm 3 (4.9 MPN/kg and 14.3%). No significant differences in occurrence and levels in fresh poultry litter samples were seen for Farm 2 (6.2 MPN/kg and 16.7%) versus Farm 1 or 3. *Salmonella* was only detected once from stored poultry litter samples during this study (October 2013, broiler Farm 1) (Figure 4A).

**Figure 5:**
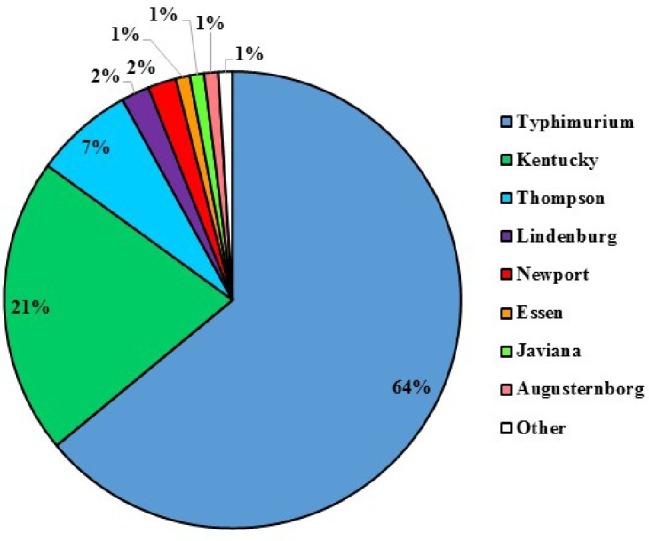
Serovar diversity of total identified *S. enterica* isolated from poultry litter samples collected from all three tested commercial broiler Farms 1–3 on the ESV (*n* = 210).

Among the 210 confirmed *Salmonella* isolates that were recovered from poultry litter samples, a total of eight serotypes were identified by molecular serotyping. Typhimurium was the most frequent serotype (64%) recovered from poultry litter samples, followed by Kentucky (21%). Serotype Newport represented 2% of the strains isolated from poultry litter samples (Figure 5).

### *Salmonella* Prevalence and Diversity in Field Soils Amended With Poultry Litter

**Figure 6:**
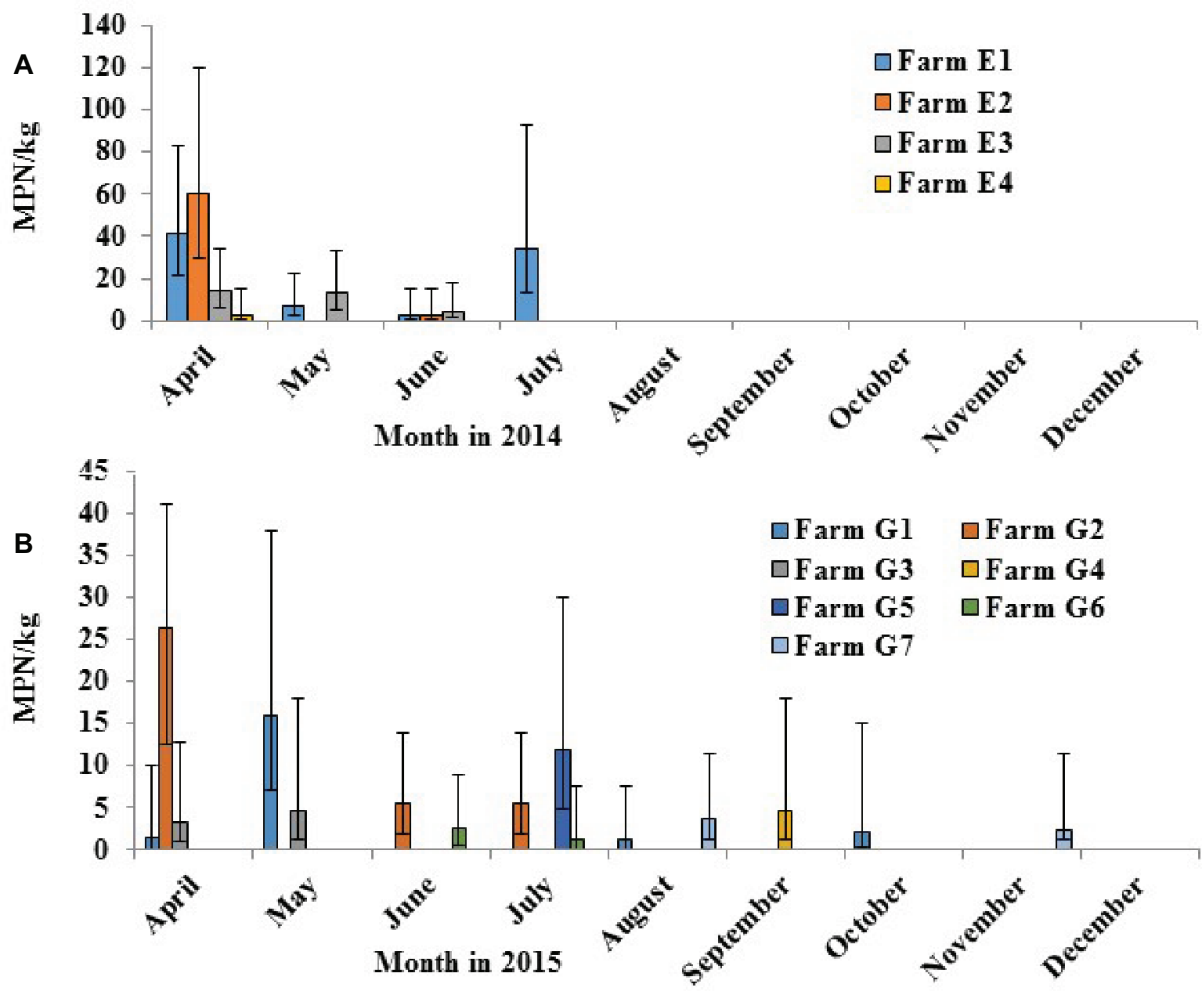
Prevalence of *Salmonella* in field soils in 2014 [**(A)** tested positive in 4 of 10 crop farms with poultry litter fertilization in April] and in 2015 [**(B)** positive in the 7 of 14 farms with poultry litter fertilization in April (G1–G3) or in May (G4–G7)].

*Salmonella* was detected in four of the 10 crop farms (E1–E10) applied with poultry litter in 2014 (Figure 6A). Decreased levels of *Salmonella* over time from April to July from poultry litter amended soil samples were observed from the four *Salmonella* positive farms (E1, E2, E3, and E4), ranging from 60 to 2.2 MPN/kg. *Salmonella* was shown to survive up to 4 months in Farm E1. The most frequent serotypes identified by molecular serotyping from poultry litter amended soil samples in 2014 were Typhimurium (38%), Norwich (33%), and Kentucky (21%).

**Figure 7:**
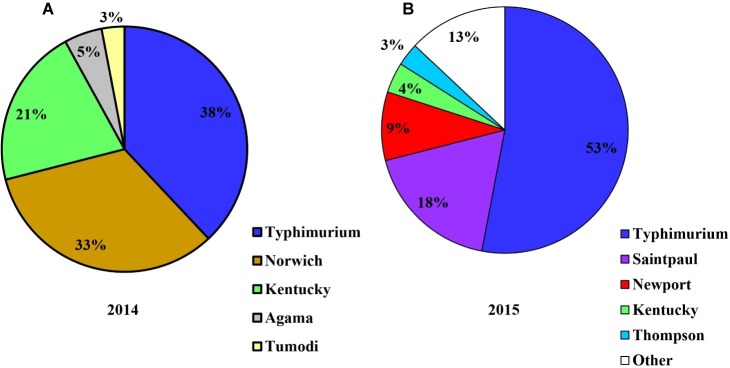
Diversity of *S. enterica* serovars isolated from field soils of conventional farms applied with poultry litter on the ESV in 2014 [**(A)**, *n* = 40] and in 2015 [**(B)**, *n* = 100].

In 2015, *Salmonella* was recovered in seven of the 14 crop farms (G1–G14) after poultry litter application (Figure 6B). Similar to 2014, *Salmonella* levels from poultry litter amended soil decreased over time, ranging from 26.3 to 1.47 MPN/kg. *Salmonella* was observed to survive up to 7 months in Farms G1 and G7. A total of 100 confirmed *Salmonella* isolates were recovered from poultry litter amended soil samples in 2015. Typhimurium (53%, Figure 7) was the most frequent serotype identified by molecular serotyping.

## Discussion

In this study, *Salmonella* population, diversity and dynamics were examined from various water sources, poultry litter, and field soils in commercial farms on the ESV. Based on the results, *Salmonella* was most prevalent in the samples tested from bay water, followed by creek, pond, and well water. However, higher *Salmonella* population density was found in the creek water samples. The average prevalence and population density of *Salmonella* in surface water on the ESV were lower, compared to the results of previous studies performed in Florida (McEgan et al., 2013; Topalcengiz et al., 2017), but similar to the studies conducted in Georgia (Luo et al., 2016) and in the ESV during the following years (Truitt et al., 2018), which indicates that the prevalence and distribution of *S. enterica* may vary at different locations in the same geographic region.

*Salmonella* was isolated from three of the four wells tested. The wells in this region are relatively shallow with an average depth of less than 60 m. Waste can enter the ground water through different mechanisms, including sewage overflow, improperly working sewage systems, contaminated storm water runoff, and agricultural runoff, which may cause the pollution of wells especially after flooding (Centers for Disease Control and Prevention, 2015). It is noteworthy that Pond D had the highest *Salmonella* MPN values, and Well D (located from Farm D) had the largest prevalence ratio, compared to the other ponds and wells tested. The relatively high levels of *Salmonella* in Farm D may be a result of distinctive agricultural practices and environmental conditions; for example, there was a significant correlation between *Salmonella* population and water turbidity in tested pond water samples in 2015 (*p* = 0.013, data not shown).

An increase of *Salmonella* was observed in pond water samples in April (2014) and March (2015), which might be associated with an increase in temperature (Supplementary Figure S3), rainfall (Supplementary Figure S4), and the application of poultry litter at this period of the year in the region. There was an obvious yearly difference for *Salmonella* serovar diversity in both pond and well irrigation water samples. *Salmonella* Newport percentage decreased from 40% in 2014 to 33% in 2015, while the percentage of Thompson increased from 6 to 28%. Interestingly, Newport was the dominant serovar in isolates from well water in 2014 (65%), but the percentage dropped to 12% in 2015. The exact cause of the shifting in *Salmonella* serovar diversity is unclear. Weather conditions, agricultural practices, phenotypic and genomic characteristics of various *Salmonella* strains may aid in the transmission and survival in agricultural environments. The limitation of sample size due to the low population density and scattered distribution of this foodborne pathogen in the environment may also result in the observed difference. A following study about microbial quality of pond water in the ESV reported that the percentage of Newport in total identified *Salmonella* isolates was 50% in 2015 and 28% in 2016 (Truitt et al., 2018). Distribution, population, and diversity of *Salmonella* spp. varies among different types of water samples and between the 2 years in this study. Further survival comparison studies and genetic analysis to compare different strains isolated from this agricultural area, such as strains of serovars Newport and Typhimurium, can benefit the identification of specific bacterial features that contribute to colonization and survival variances of *Salmonella* in different agricultural environments.

*Salmonella* Typhimurium, Kentucky, and Thompson were identified as the predominate serovars in fresh poultry litter as well as in poultry litter amended field soils, which is consistent with former reports (Linares et al., 1984; Skov et al., 1999; Ibrahim et al., 2013; Raufu et al., 2014). In contrast to the high ratio of *Salmonella* Newport in isolates from water samples, the percentage of *Salmonella* Newport in isolates from poultry litter and poultry litter amended soil samples on the ESV was significantly lower, which is in concurrence with a former study conducted in this region (Bell et al., 2015). The absence or low proportion of *Salmonella* Newport in poultry litter and field soil samples may indicate that the broiler farms and management practices around the application of poultry litter for the ESV region may not be the direct source of *Salmonella* Newport contamination that resulted in several salmonellosis outbreaks (Centers for Disease Control and Prevention, 2005, 2007; Greene et al., 2008; Bennett et al., 2015).

The distinct *Salmonella* serovar diversity in water, poultry litter, and poultry litter amended soil samples indicates the persistence of specific serovars, for example in water sources in the study reported here (*Salmonella* Newport). Furthermore, the re-introduction of *Salmonella* in birds and deer that live in the region (Gruszynski et al., 2014a) may play an important role in the epidemiology cycle. Future studies may include surveys of wildlife in the region as wildlife (specifically avian or reptile populations) may be a possible contamination source of *Salmonella* Newport (Gruszynski et al., 2014b).

Culture-dependent methods were used for *Salmonella* enumeration in this study, which may lead to underestimation of bacterial populations due to the limitation in detection of viable but non-culturable cells. Further culture-independent methods and genomic analyses using whole genome sequencing could be performed to compare the typical strains in various samples and identify specific genetic traits contributing to bacterial survival in different environment niches. In addition, the physical, chemical, and biological factors in different environments may impact the colonization and persistence of *Salmonella* by performing a screen/selective function for *Salmonella*. Further studies on microbial community analysis of different environmental (e.g., agricultural, natural) samples will provide information to better understand the synergistic, antagonistic, and/or symbiotic interactions of certain species with *Salmonella* that may consequently cause the variance of *Salmonella* serovar diversity and persistence.

As reported in our previous study (Gu et al., 2018), naturally contaminated irrigation water and fertilizer (poultry litter) were potential sources of *Salmonella* in tomato fields. The persistence of *Salmonella* in experimental plots (detectable during 4-month growth season) and commercial crop fields (4–6 months) was comparable. Agricultural practices and bacterial genetic traits (among different *Salmonella* serotypes) may play important roles in the likelihood of *Salmonella* contamination and persistence in fields.

The results of this study provide valuable information for farmers, stakeholders, and the public on potential sources and pathways to contamination events of fresh produce. This study also provides scientific data on *Salmonella* serovar distribution and diversity in a major agricultural environment (ESV), which will inform future epidemiological and risk assessment studies on *Salmonella*, and also assist in development of mitigation strategies to limit potential contamination events.

## Data Availability Statement

The datasets for this article are not publicly available. Requests to access the datasets should be directed to Ganyu Gu, gganyu1@vt.edu.

## Author Contributions

GG and SR contributed to the study conception and design. GG, JZ, and ER contributed to the acquisition of data. GG and LS contributed to analysis and interpretation of data. GG contributed to drafting of manuscript. LS, JZ, ER, and SR contributed to critical revision.

### Conflict of Interest

The authors declare that the research was conducted in the absence of any commercial or financial relationships that could be construed as a potential conflict of interest.

## References

[ref1] Angelo K. M. Chu A. Anand M. Nguyen T. Bottichio L. Wise M. (2015). Outbreak of *Salmonella* Newport infections linked to cucumbers — United States, 2014. Morb. Mortal. Wkly Rep. 64, 144–147. Available at: https://www.cdc.gov/mmwr/preview/mmwrhtml/mm6406a3.htm?s_cid=mm6406a3_w PMC4584703 25695319

[ref2] Bell R. L. Zheng J. Burrows E. Allard S. Wang C. Y. Keys C. E. (2015). Ecological prevalence, genetic diversity, and epidemiological aspects of *Salmonella* isolated from tomato agricultural regions of the Virginia Eastern Shore. Front. Microbiol. 6:415. 10.3389/fmicb.2015.00415 25999938 PMC4423467

[ref3] Bennett S. D. Littrell K. W. Hill T. A. Mahovic M. Behravesh C. B. (2015). Multistate foodborne disease outbreaks associated with fresh tomatoes, United States, 1990–2010: a recurring public health problem. Epidemiol. Infect. 143, 1352–1359. 10.1017/S0950268814002167 25167220 PMC9507195

[ref4] Centers for Disease Control and Prevention (2005). Outbreaks of *Salmonella* infections associated with eating Roma tomatoes—United States and Canada, 2004. Can. Commun. Dis. Rep. 31, 225–228. Available at: https://www.cdc.gov/mmwr/preview/mmwrhtml/mm5413a1.htm 16669127

[ref5] Centers for Disease Control and Prevention (2007). Multistate outbreaks of *Salmonella* infections associated with fresh tomatoes eaten in restaurants --- United States, 2005-2006. Morb. Mortal. Wkly Rep. 56, 909–911. Available at: https://www.cdc.gov/mmwr/preview/mmwrhtml/mm5635a3.htm 17805221

[ref6] Centers for Disease Control and Prevention (2009). Standard protocol molecular determination of serotype in *Salmonella*, workshop on molecular determination of serotype of *Salmonella*. Atlanta, GA: Centers for Disease Control and Prevention.

[ref7] Centers for Disease Control and Prevention (2015). *Salmonella* and drinking water from private wells. Available at: https://www.cdc.gov/healthywater/drinking/private/wells/disease/salmonella.html (Accessed November 26, 2019).

[ref8] Centers for Disease Control and Prevention (2019). Reports of selected *Salmonella* outbreak investigations. Available at: https://www.cdc.gov/salmonella/outbreaks.html (Accessed July 21, 2019).

[ref9] Fitzgerald C. Collins M. van Duyne S. Mikoleit M. Brown T. Fields P. (2007). Multiplex, bead-based suspension array for molecular determination of common *Salmonella* serogroups. J. Clin. Microbiol. 45, 3323–3334. 10.1128/JCM.00025-07 17634307 PMC2045348

[ref10] Food Safety News (FSN) (2011). Norovirus, *Salmonella* key culprits in CDC outbreak report. Available at: http://www.foodsafetynews.com/2011/09/norovirus-remains-leading-cause-of-outbreaks/#.Vwfdxnpmr3A (Accessed July 21, 2019).

[ref11] Franz E. Semenov A. V. Termorshuizen A. J. de Vos O. J. Bokhorst J. G. van Bruggen A. H. C. (2008a). Manure-amended soil characteristics affecting the survival of E-coli O157:H7 in 36 Dutch soils. Environ. Microbiol. 10, 313–327. 10.1111/j.1462-2920.2007.01453.x 18199123

[ref12] Franz E. Semenov A. V. van Bruggen A. H. C. (2008b). Modelling the contamination of lettuce with *Escherichia coli* O157:H7 from manure-amended soil and the effect of intervention strategies. J. Appl. Microbiol. 105, 1569–1584. 10.1111/j.1365-2672.2008.03915.x 19146493

[ref13] Greene S. K. Daly E. R. Talbot E. A. Demma L. J. Holzbauer S. Patel N. J. (2008). Recurrent multistate outbreak of *Salmonella* Newport associated with tomatoes from contaminated fields, 2005. Epidemiol. Infect. 136, 157–165. 10.1017/S095026880700859X 17475091 PMC2870807

[ref14] Gruszynski K. Pao S. Kim C. Toney D. M. Wright K. Colón A. (2014a). Evaluating gulls as potential vehicles of *Salmonella enterica* serotype Newport (JJPX01.0061) contamination of tomatoes grown on the eastern shore of Virginia. Appl. Environ. Microbiol. 80, 235–238. 10.1128/AEM.02809-13 24141129 PMC3911029

[ref15] Gruszynski K. Pao S. Kim C. Toney D. M. Wright K. Ross P. G. (2014b). Evaluating wildlife as a potential source of *Salmonella* serotype Newport (JJPX01.0061) contamination for tomatoes on the eastern shore of Virginia. Zoonoses Public Health 61, 202–207. 10.1111/zph.12061 23773825

[ref16] Gu G. Ottesen A. Zheng J. Oryang D. O. Boyer R. Strawn L. K. (2016). “Diversity and dynamics of *Salmonella enterica spp. in* irrigation water and poultry litter amended fields on the eastern shore of Virginia” in *IAFP annual meeting*.

[ref17] Gu G. Strawn L. K. Oryang D. Zheng J. Reed E. Ottesen A. (2018). Agricultural practices influence *Salmonella* contamination and survival in pre-harvest tomato production. Front. Microbiol. 9:2451. 10.3389/fmicb.2018.02451 30386314 PMC6198144

[ref18] Hintz L. D. Boyer R. R. Ponder M. A. Williams R. C. Rideout S. R. (2010). Recovery of *Salmonella enterica* Newport introduced through irrigation water from tomato (*Lycopersicum esculentum*) fruit, roots, stems, and leaves. HortScience 45, 675–678. 10.21273/HORTSCI.45.4.675

[ref19] Hurley A. H. Roscoe M. E. (1983). Automated statistical analysis of microbial enumeration by dilution series. J. Appl. Bacteriol. 55, 159–164.

[ref20] Ibrahim M. A. Emeash H. H. Ghoneim N. H. Abdel-Halim M. A. (2013). Seroepidemiological studies on poultry Salmonellosis and its public health importance. J. World Poult. Res. 3, 18–23. Available at: https://pdfs.semanticscholar.org/dc81/6dc64803a4425e3986541a0c78b11965101b.pdf?_ga=2.264315599.1907830852.1575420336-1749248137.1575420336

[ref21] Islam M. Morgan J. Doyle M. P. Phatak S. C. Millner P. Jiang X (2004a). Persistence of *Salmonella enterica* serovar typhimurium on lettuce and parsley and in soils on which they were grown in fields treated with contaminated manure composts or irrigation water. Foodborne Pathog. Dis. 1, 27–35. 10.1089/153531404772914437 15992259

[ref22] Islam M. Morgan J. Doyle M. P. Phatak S. C. Millner P. Jiang X. (2004b). Fate of *Salmonella enterica* serovar Typhimurium on carrots and radishes grown in fields treated with contaminated manure composts or irrigation water. Appl. Environ. Microbiol. 70, 2497–2502. 10.1128/aem.70.4.2497-2502.2004 15066849 PMC383101

[ref24] Linares A. P. Cohen S. H. Goldstein E. Kelley A. D. K. Eisenstein T. K. (1984). Febrile gastroenteritis due to *Salmonella thompson*—report of an outbreak. West. J. Med. 141, 203–205. 6495726 PMC1021737

[ref25] Luo Z. Gu G. Ginn A. van Bruggen A. H. C. Danyluk M. Wright A. (2016). Distribution and characterization of *Salmonella enterica* isolates from irrigation ponds in southeastern USA. Appl. Environ. Microbiol. 81, 8243–8253. 10.1128/AEM.04086-14 PMC4475880 25911476

[ref26] Luo Z. Gu G. Giurcanu M. C. Adams M. Vellidis G. van Bruggen A. H. C. (2014). Development of a novel cross-streaking method for isolation, confirmation, and enumeration of *Salmonella* from irrigation ponds. J. Microbiol. Methods 101, 86–92. 10.1016/j.mimet.2014.03.012 24732066

[ref27] McEgan R. Mootian G. Goodridge L. D. Schaffner D. W. Danyluk M. D. (2013). Predicting *Salmonella* populations from biological, chemical, and physical indicators in Florida surface waters. Appl. Environ. Microbiol. 79, 4094–4105. 10.1128/AEM.00777-13 23624476 PMC3697547

[ref28] McQuiston J. R. Waters R. J. Dinsmore B. A. Mikoleit M. L. Fields P. I. (2011). Molecular determination of H antigens of *Salmonella* by use of a microsphere-based liquid array. J. Clin. Microbiol. 49, 565–573. 10.1128/JCM.01323-10 21159932 PMC3043481

[ref29] Natvig E. E. Ingham S. C. Ingham B. H. Cooperband L. R. Roper T. R. (2002). *Salmonella enterica* serovar Typhimurium and *Escherichia coli* contamination of root and leaf vegetables grown in soils with incorporated bovine manure. Appl. Environ. Microbiol. 68, 2737–2744. 10.1128/AEM.68.6.2737-2744.2002 12039728 PMC123957

[ref30] Ohtomo R. Minato K. Saito M. (2004). Survival of *Escherichia coli* in a field amended with cow feces slurry. Soil Sci. Plant Nutr. 50, 575–581. 10.1080/00380768.2004.10408514

[ref31] Opara O. O. Carr L. E. Russek-Cohen E. Tate C. R. Mallinson E. T. Miller R. G. (1992). Correlation of water activity and other environmental conditions with repeated detection of *Salmonella* contamination on poultry farms. Avian Dis. 36, 664–671. 1417596

[ref32] Park S. Szonyi B. Gautam R. Nightingale K. Anciso J. Ivanek R. (2012). Risk factors for microbial contamination in fruits and vegetables at the preharvest level: a systematic review. J. Food Prot. 75, 2055–2081. 10.4315/0362-028X.JFP-12-160 23127717

[ref33] Pope M. J. Cherry T. E. (2000). An evaluation of the presence of pathogens on broilers raised on poultry litter treatment-treated litter. Poult. Sci. 79, 1351–1355. 10.1093/ps/79.9.1351 11020084

[ref34] Raufu I. A. Fashae K. Ameh J. A. Ambali A. G. Ogunsola F. T. Coker A. O. (2014). Persistence of fluoroquinolone-resistant *Salmonella enterica* serovar Kentucky from poultry and poultry sources in Nigeria. J. Infect. Dev. Ctries. 8, 384–388. 10.3855/jidc.3495 24619272

[ref35] Semenov A. V. van Overbeek L. van Bruggen A. H. C. (2009). Percolation and survival of *Escherichia coli* O157:H7 and *Salmonella enterica* serovar Typhimurium in soil amended with contaminated dairy manure or slurry. Appl. Environ. Microbiol. 75, 3206–3215. 10.1128/AEM.01791-08 19270130 PMC2681632

[ref36] Skov M. N. Carstensen B. Tornoe N. Madsen M. (1999). Evaluation of sampling methods for the detection of *Salmonella* in broiler flocks. J. Appl. Microbiol. 86, 695–700. 10.1046/j.1365-2672.1999.00715.x 10212414

[ref37] Solomon E. B. Yaron S. Matthews K. R. (2002). Transmission of *Escherichia coli* O157:H7 from contaminated manure and irrigation water to lettuce plant tissue and its subsequent internalization. Appl. Environ. Microbiol. 68, 397–400. 10.1128/aem.68.1.397-400.2002 11772650 PMC126537

[ref38] Strawn L. K. Gröhn Y. T. Warchocki S. Worobo R. W. Bihn E. A. Wiedmann M. (2013). Risk factors associated with *Salmonella* and *Listeria monocytogenes* contamination of produce fields. Appl. Environ. Microbiol. 80, 3982–3991. 10.1128/AEM.02831-13 PMC3837806 24077713

[ref39] Suresh T. Hathab A. A. M. Harshac H. T. Lakshmanaperumalsamya P. (2011). Prevalence and distribution of *Salmonella* serotypes in marketed broiler chickens and processing environment in Coimbatore City of southern India. Food Res. Int. 44, 823–825. 10.1016/j.foodres.2011.01.035

[ref40] Topalcengiz Z. Strawn L. K. Danyluk M. D. (2017). Microbial quality of agricultural water in Central Florida. PLoS One 12:e0174889. 10.1371/journal.pone.0174889 28399144 PMC5388333

[ref41] Truitt L. N. Vazquez K. M. Pfuntner R. C. Rideout S. L. Havelaar A. H. Strawn L. K. (2018). Microbial quality of agricultural water used in produce preharvest production on the Eastern Shore of Virginia. J. Food Prot. 81, 1661–1672. 10.4315/0362-028X.JFP-18-185 30212229

[ref42] Van Asselt E. D. Thissen J. T. N. M. van der Fels-Klerx H. J. (2009). *Salmonella* serotype distribution in the Dutch broiler supply chain. Poult. Sci. 88, 2695–2701. 10.3382/ps.2009-00074 19903970

[ref43] Virginia Department of Environmental Quality (VDEQ) (2010). Requirements for poultry litter use and storage. Available at: https://www.deq.virginia.gov/Portals/0/DEQ/Water/VirginiaPollutionAbatement/PWMForms/Poultry_Litter_Fact_Sheet_rev_12.2010.pdf (Accessed July 21, 2019).

[ref44] You Y. Rankin S. C. Aceto H. W. Benson C. E. Toth J. D. Dou Z. (2006). Survival of *Salmonella enterica* serovar Newport in manure and manure-amended soils. Appl. Environ. Microbiol. 72, 5777–5783. 10.1128/AEM.00791-06 16957193 PMC1563654

