# Supplementary material for: Diversity and Dynamics of Salmonella enterica in Water Sources, Poultry Litters, and Field Soils Amended With Poultry Litter in a Major Agricultural Area of Virginia
Source: Front Microbiol. 2019 Dec 17;10:2868. doi: 10.3389/fmicb.2019.02868 (PMC6951424; doi:10.3389/fmicb.2019.02868)

Figure S1. Monthly variance of *Salmonella* prevalence (A) and population density (B) during sampling periods of 2014 and 2015. Bars represent standard errors.

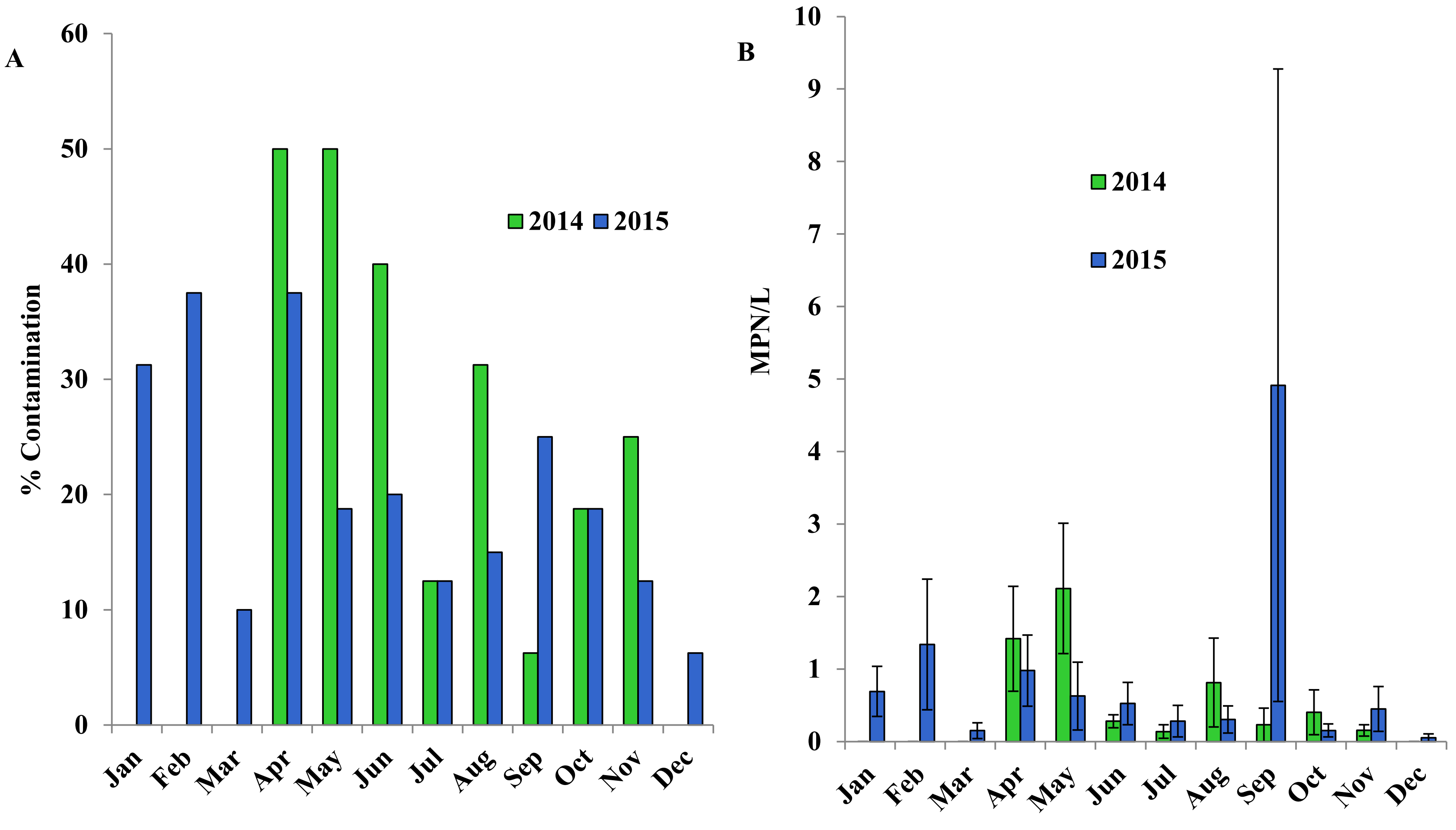

Figure S2. Prevalence and MPN values of *S. enterica* spp. in creek water samples at farm B.

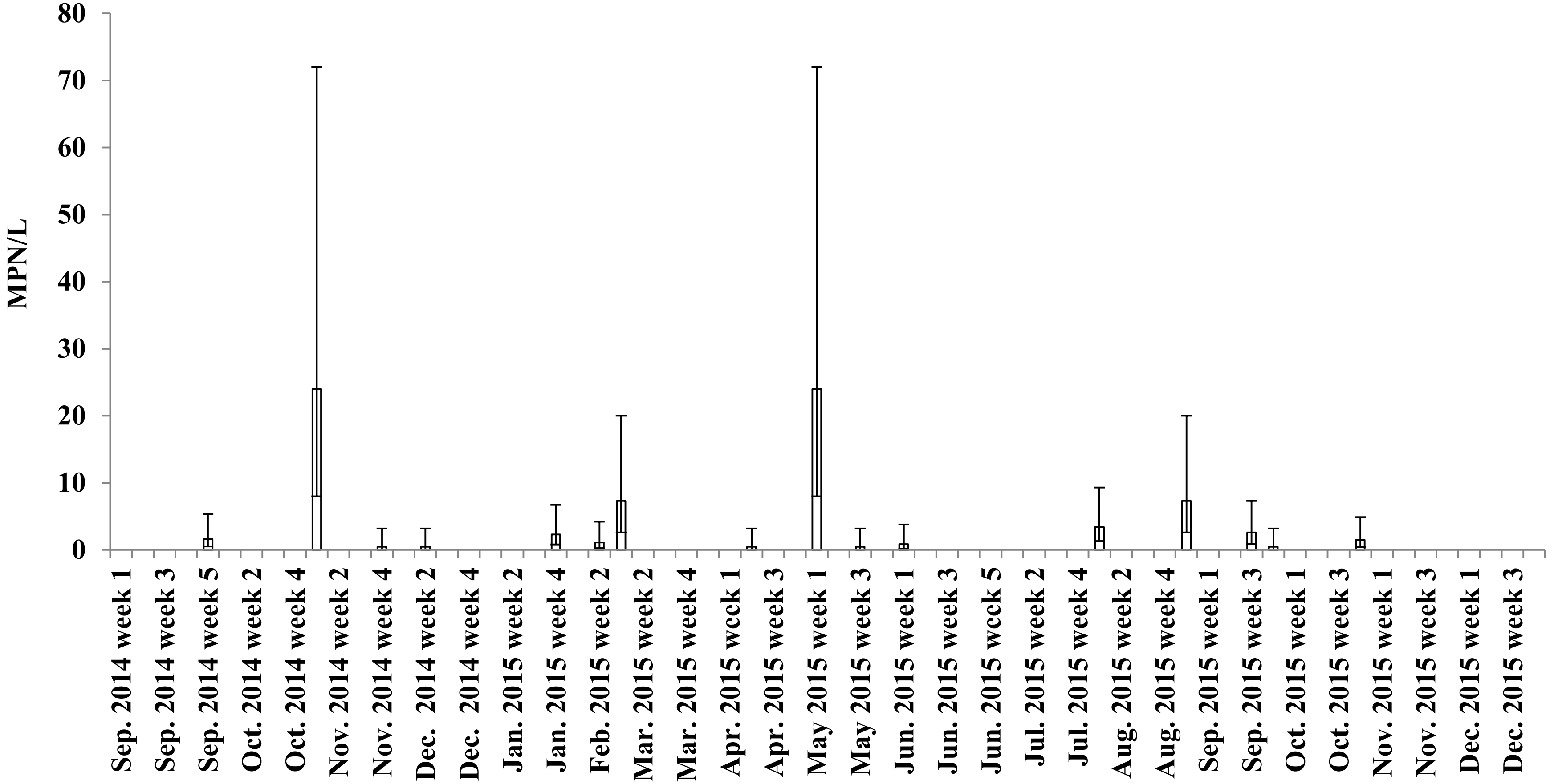

Figure S3. Dynamics of temperature of tested farms in 2014 (A) and 2015 (B).

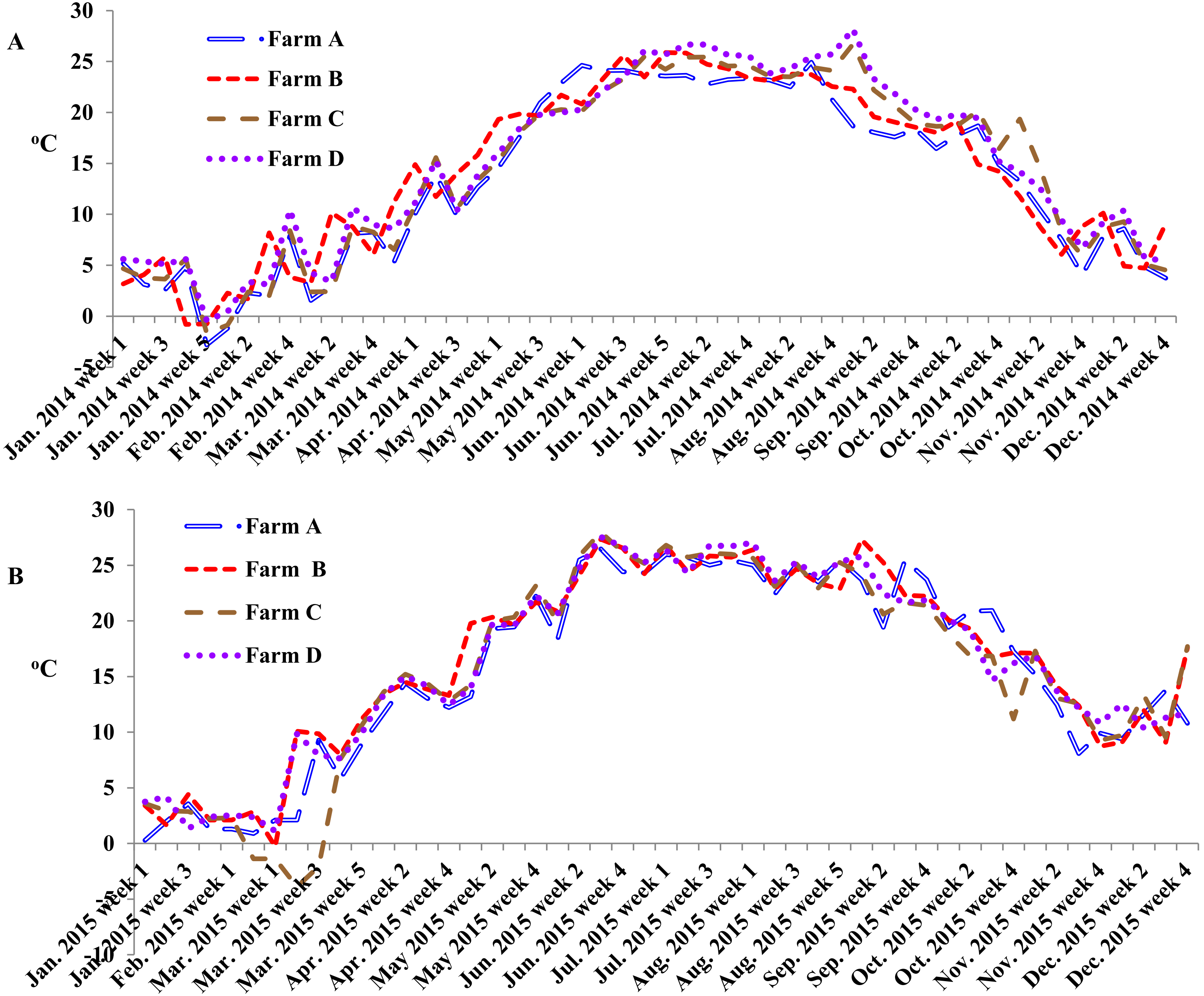

Figure S4. Dynamics of total rainfall of tested farms in 2014 (A) and 2015 (B).

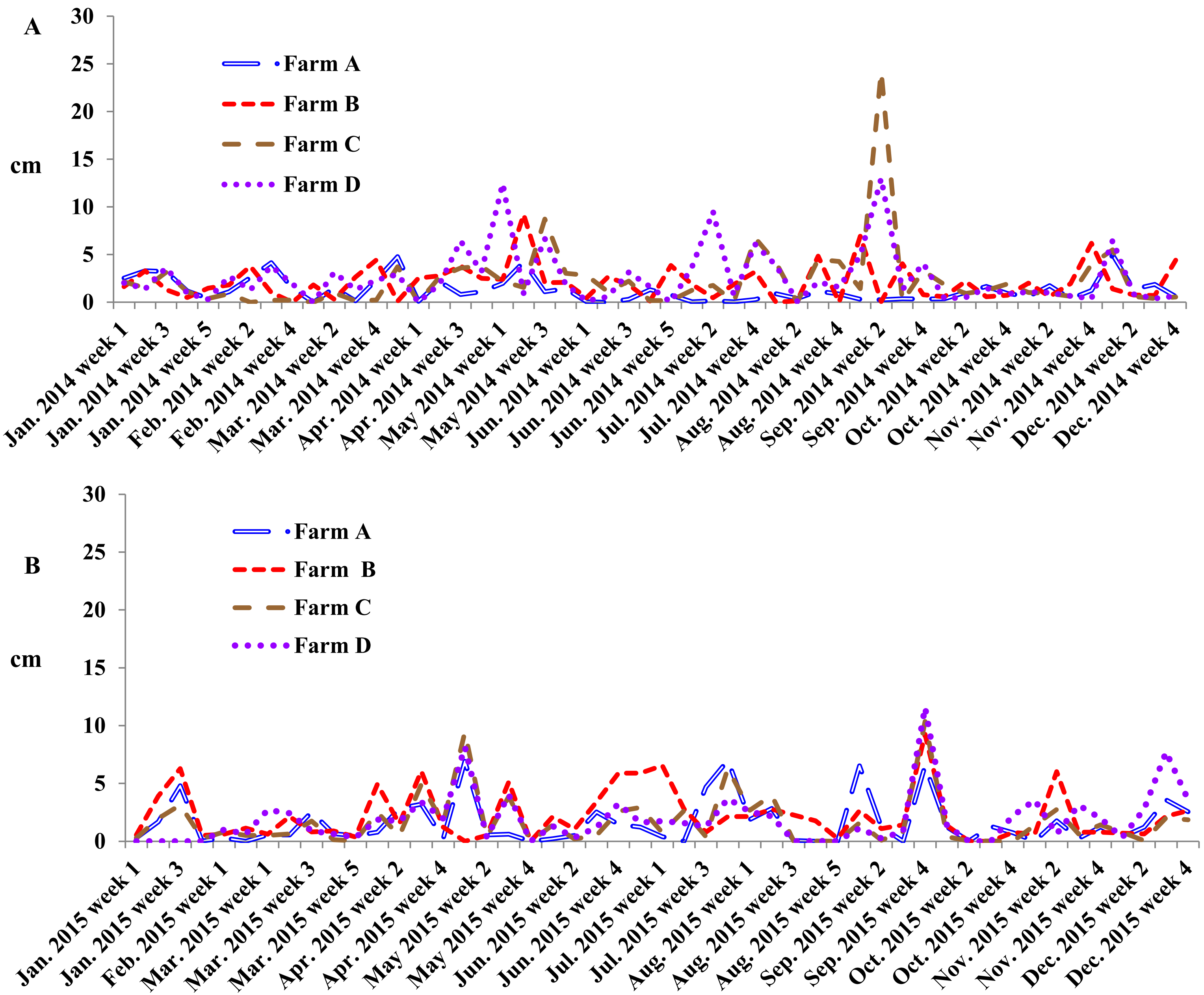

Supplement: Supplementary file 1 [file Data_Sheet_1.PDF]
